# A Review of COVID-19 Mass Testing in the United Arab Emirates

**DOI:** 10.3389/fpubh.2021.661134

**Published:** 2021-05-12

**Authors:** Farida Al-Hosani, Shereena Al-Mazrouei, Shammah Al-Memari, Zain Al-Yafei, Marília Silva Paulo, Erik Koornneef

**Affiliations:** ^1^Abu Dhabi Public Health Center, Abu Dhabi, United Arab Emirates; ^2^Abu Dhabi Health Services Company – SEHA, Abu Dhabi, United Arab Emirates; ^3^College of Medicine & Health Sciences, Institute of Public Health, UAE University, Al Ain, United Arab Emirates

**Keywords:** COVID-19, mass testing strategy, United Arab Emirates, public health policy, public health strategy, SARS-CoV-2

## Abstract

Appropriate diagnostic testing to identify persons infected with SARS-COV-2 is a vital part of a health system's ability to control the global pandemic of COVID-19 disease. The primary purpose of this review is to provide an overview of the mass testing strategy implemented throughout the UAE and the overall impact it has made on containing and controlling the spread of the disease. This study describes the mass testing strategy and capacity of the UAE during the pandemic of the new coronavirus SARS-COV-2. The UAE has conducted 15 million polymerase chain reaction (PCR) tests to SARS-COV-2, as of 15 November 2020. The number of tests per day varied from 10,000 by the end of March to 120,000 tests per day in November 2020. The mass testing initiative across the entire UAE forms an integral part of a bigger strategy focusing on testing, tracing contacts and isolating positive cases.

## Introduction

Since the first identification of a cluster of respiratory illnesses caused by a new coronavirus in late 2019, the highly contagious disease, COVID-19, has rapidly spread across the globe. The first case of the new coronavirus SARS-COV-2 disease was detected in the United Arab Emirates (UAE) on 29 January 2020 (1). Since the first reported case of COVID-19, the UAE government has implemented a strategy to contain the disease, prevent its spread and treat infected patients requiring medical care (2). The World Health Organization (WHO) officially declared COVID-19 a pandemic on 11 March 2020 (3).

Appropriate diagnostic testing to identify persons infected with SARS-COV-2 is a vital part of a health system's ability to control the global pandemic of COVID-19 disease. The mass testing initiative across the entire UAE is part of a bigger strategy focusing on testing, tracing contacts and isolating positive cases. The UAE's strategy includes a range of public health interventions, including mobile drive-through and travel restrictions (4). Other interventions included the closure of schools, public amenities, and curfews during March and April 2020 (4).

According to the Oxford Covid-19 Government Response Tracker (OxCGRT) the UAE government's testing policy can be classified as “open public testing, (e.g., “drive-through” testing available to asymptomatic people)” (5). The UAE adopted this open public testing policy in early April. Other countries with a similar open public testing policy are Canada, China, Germany, Saudi Arabia, and South Korea (6).

The UAE has seven Emirates that collaborate to deliberately plan the development of their society to sustain and strengthen national unity, promote continuous economic growth and personal health and well-being (7). There are three main authorities responsible for overseeing the UAE health system: the Department of Health in the Emirate of Abu Dhabi, the Dubai Health Authority, and the Ministry of Health and Prevention, responsible for the remaining five Emirates. The latter two also provide public health services in their respective geographical areas. The Emirate of Abu Dhabi separated the provision of services from the regulatory, oversight functions in 2007, with a strong focus on providing all residents access to high quality, affordable world-class health services (8).

According to the World Bank, the UAE had a population of 9.68 million in 2019 (9). The population of the UAE is relatively young, with a median age of 30, but amongst UAE nationals, who account for ~11% of the population, 79% are aged <35 years old (10). The population of the UAE increased from 5% of annual growth in 2000 to 15% in 2007 (11) due to the natural growth of nationals but also due to the recruitment of expatriates to work in various industries, including the service and construction sector (12). Accompanying these changes in population, the number of healthcare facilities, including hospitals has also increased dramatically over the same years in the UAE. One study reported a doubling of hospital beds in ten-years (2005–2014), accompanied by a 5-fold increase in physician numbers (10).

Since the beginning of the COVID-19 outbreak, the responsible health regulatory authorities in the UAE, together with the main providers and statutory agencies such as the National Crisis and Emergency Management Authority (NCEMA) prepared, published and implemented a unified set of National Guidelines for Clinical Management and Treatment of COVID-19 (13). The UAE guidelines reflect international guidance, in particular from the WHO. Whilst WHO does not specifically recommend mass testing programs, the WHO guidance (14) asserts that testing of probable cases, where resources allow, may be useful since it can exclude patients as cases and reduce the burden required to isolate and contact trace those patients. In line with WHO recommendations (15), the UAE has applied polymerase chain reaction (PCR) tests to detect the presence of SARS-CoV-2 RNA as the basis for COVID-19 case confirmation. By law, all clinical laboratories in the UAE are required to acquire accreditation from relevant accreditation bodies (16).

One of the main objectives of the UAE COVID-19 plan is to ensure fully accessible testing and treatment services for all residents, regardless of insurance coverage (13). To assist the tracking and cases tracing process, the healthcare regulatory authorities in the country launched a free application (ALHOSN UAE) that enables users to exchange anonymized IDs stored in an encrypted form so that their health authorities can easily contact individuals at risk. The application also warns users when an infected person is nearby, thereby preventing possible infection (17).

The process of isolating positive cases is outlined in the unified guidelines: once a case is confirmed positive the person is quarantined in a local hotel, hospital or at home, with appropriate monitoring. At the start of the outbreak, most new positive cases were admitted to a licensed, designated healthcare facility and as the situation worsened home, and hotel quarantine became more in use for asymptomatic cases and patients with mild symptoms. During March, April and May 2020, the Abu Dhabi Healthcare Company (SEHA) treated 9,390 patients in their hospitals (13). The majority of cases (92%) were treated for short periods of time in regular wards at designated SEHA hospitals, or in quarantine hotels (13). Another large provider of healthcare services in Abu Dhabi, the Cleveland Clinic, treated 690 patients COVID-19 patients during the same 3 months in 2020 (18).

The overall aim of the UAE COVID-19 strategy is to achieve a substantial reduction in the number of cases by effective control public health interventions, including mass testing. The primary purpose of this review is to provide an overview of the mass testing strategy implemented throughout the UAE and the overall impact it has made on containing the spread of the disease.

## Materials and Methods

We conducted a cross-sectional review from open-access databases. We retrieved COVID-19 related data from the website “Our World in Data” (19). This website is a collaborative effort between researchers at the University of Oxford and Global Change Data, a non-profit organization. Our World in Data uses a variety of official national and international resources related to the COVID-19 pandemic, including the online repository and online interactive dashboard, hosted by the Center for Systems Science, and Engineering (CSSE) at Johns Hopkins University, Baltimore, MD, USA (20). It has documented COVID-19 data relating to case numbers, infection fatality rates, as well as test numbers and hospitalization rates from more than 200 countries.

Information regarding the policy responses was sourced from the Oxford Coronavirus Government Response Tracker (OxCGRT). This resource is published by researchers at the Blavatnik School of Government at the University of Oxford (6). OxCGRT collects publicly available information on 17 indicators of government responses, spanning containment and closure policies (such as school closures and restrictions in movement); economic policies; and health system policies (such as testing regimes).

## Results

The merits of testing strategies have been debated at length, with some experts promoting mass testing (21) and others arguing against mass testing due to the increased burden on the health system*s*. For example, the WHO recommends testing for all suspected cases, not necessarily testing of asymptomatic cases (14). Considering the growth in the number of hospital beds and clinicians in the UAE over the last decade, the UAE government decided to embark on mass testing, with appropriate isolation processes in place. Mass testing forms an important pillar of the UAE's strategy to contain the spread of the COVID-19 virus (4).

In terms of medical treatment of COVID-19 positive cases, the UAE government invested in private and public initiatives to increase the number of beds. In the Emirate of Abu Dhabi, the government established a field hospital in April 2020 (22), supplying for an additional 1,200 patients. Around the same time, the Emirate of Dubai announced its capacity to ramp up to additional 5,000 or more beds in field hospitals, in required, in addition to the 4,000 beds in regular, existing hospitals (23).

The largest governmental health care provider in the Emirate of Abu Dhabi, SEHA, played a pivotal role in setting up the mass testing program throughout the seven Emirates. Its first drive-through screening center was officially opened 29 of March 2020 (24). The COVID-19 test was initially offered free to priority groups (people with symptoms, senior citizens, and vulnerable groups), and later to other groups providing essential services, such as teachers and healthcare professionals. For the wider community, for those who preferred to do the examination for reassurance, the testing procedure initially cost around 100 USD. Over time, the price of the test was gradually reduced to 40 USD in October 2020.

The Abu Dhabi Healthcare Company currently operates 23 drive-through centers across the UAE. The Dubai Health Authority operates an additional number of test centers in the emirate of Dubai. In addition to this, testing is also offered by private healthcare organizations across the country.

**Figure 1:**
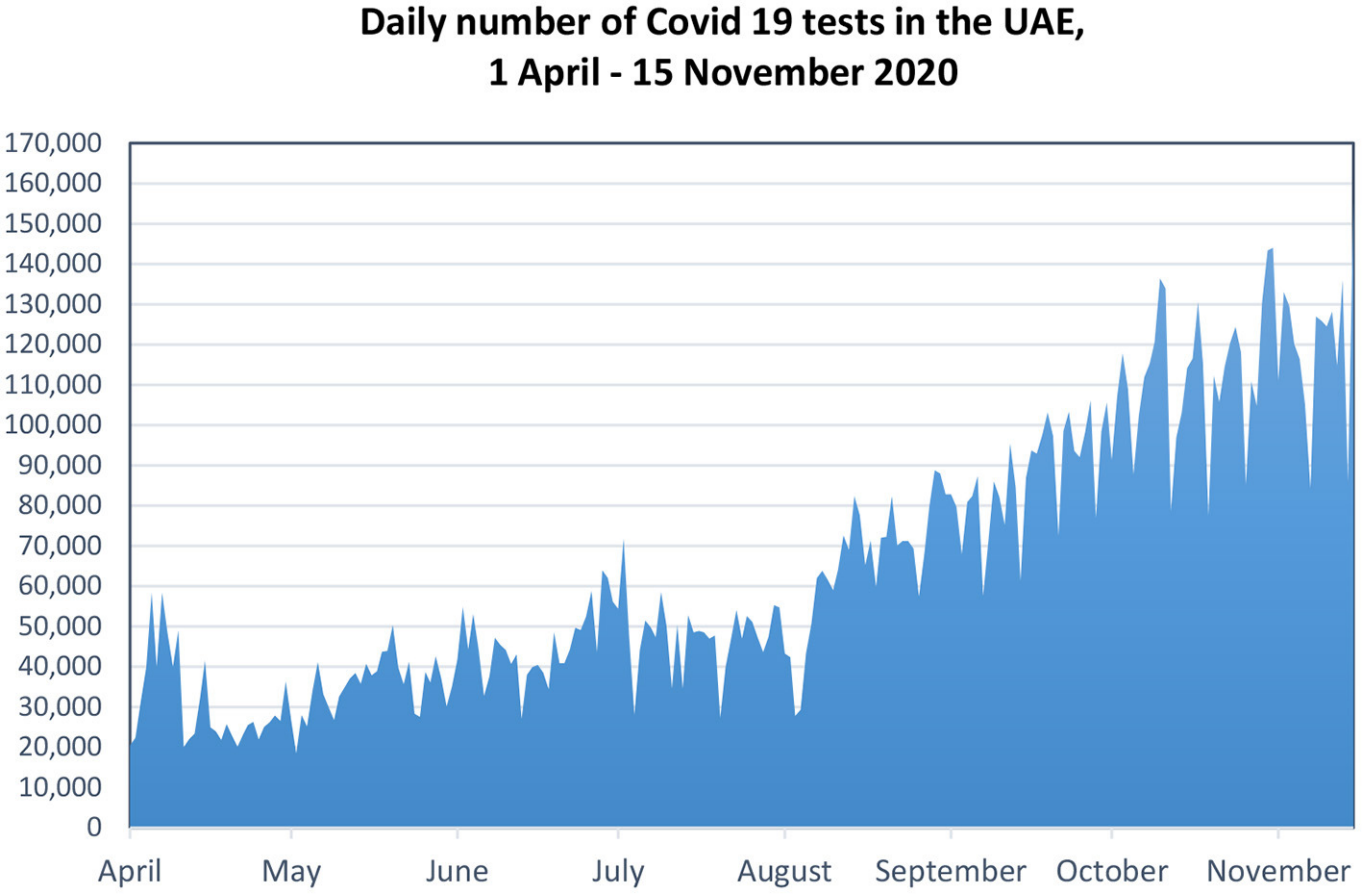
Daily number of COVID-19 tests in the UAE. Source: UAE Federal Competitiveness and Statistics Authority (FCSA).

As the figure below indicates, the daily number of COVID-19 tests increased dramatically from <10,000 in March 2020 to 120,000 in November 2020 (Figure 1). The highest number of tests was reached on 15 November 2020 when 154,882 tests were carried out in the UAE. As of 15 November 2020, a total of 15 million PCR tests were conducted in the UAE, yielding over 150,345 COVID-19 positive cases (25). From November 2020 around 100,000–140,000 tests per day are being carried out by national central laboratories and private laboratories.

The first deaths due to COVID-19 occurred on 21 March 2020, since these first reported deaths the number of tests increased from around 10,000 by the end of March to over 110,000 tests per day in October 2020. The average daily tests had reached to almost 120,000 tests per day in November, see Table 1.

**Table 1:** Average number of daily COVID-19 PCR tests, United Arab Emirates, 2020.

**Average number of daily COVID-19 PCR tests**	
Mar-20	6,357
Apr-20	30,846
May-20	35,157
Jun-20	45,253
Jul-20	47,857
Aug-20	65,106
Sep-20	87,002
Oct-20	112,240
Nov-20	119,713

*Source: UAE Federal Competitiveness and Statistics Authority (FCSA)*.

**Figure 2:**
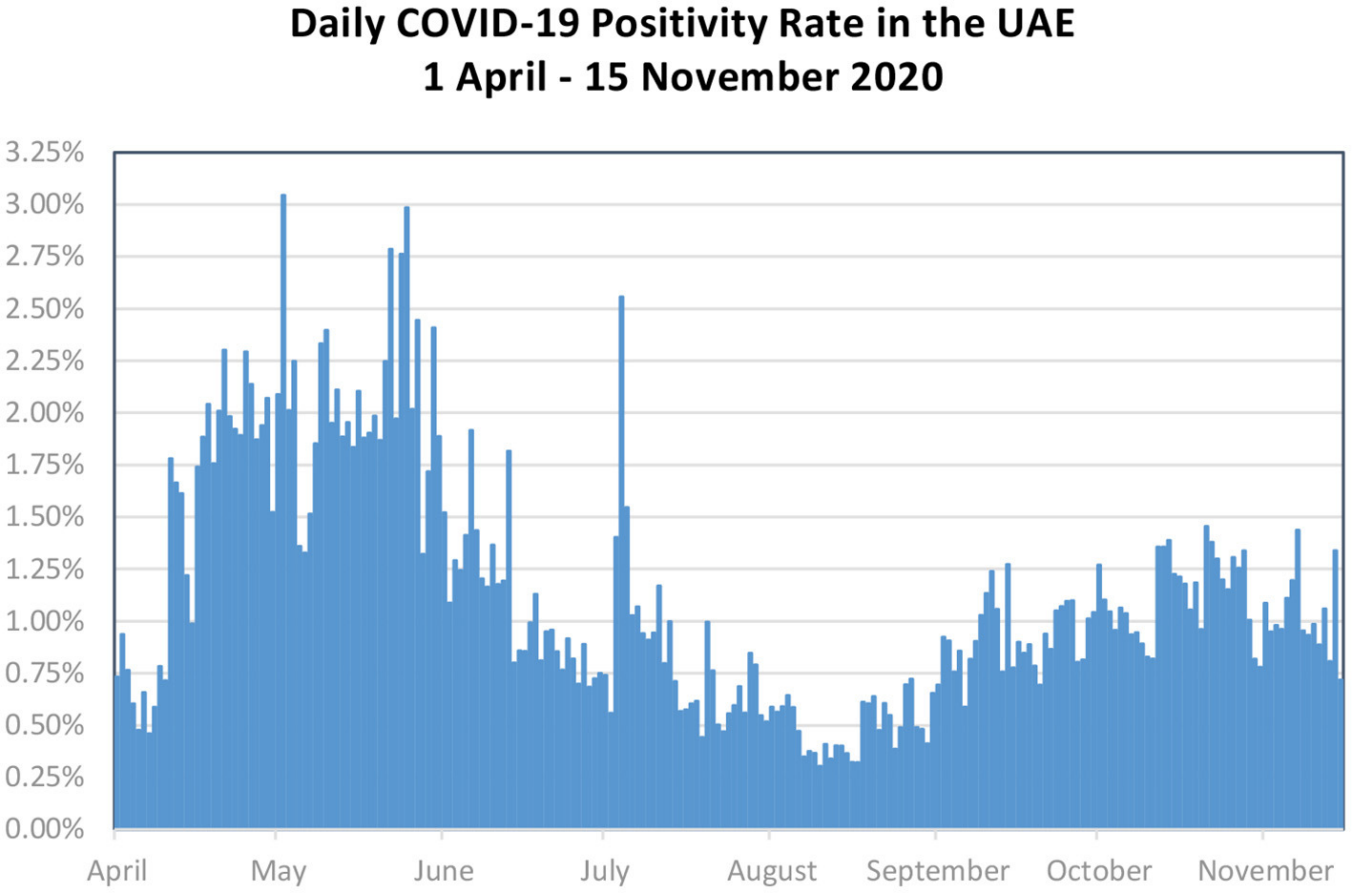
Daily COVID-19 positivity rate in the UAE. Source: UAE Federal Competitiveness and Statistics Authority (FCSA).

The percentage of all daily COVID-19 tests performed that are actually positive (positivity rate) has remained relatively stable since the beginning of April, between 0.30% on 10 of August 2020 and 3.04% on 2 of May 2020, as shown in Figure 2. It is also possible to observe a small rise in the positivity rate from August-September which can be partially explained by the worldwide second wave of COVID-19 pandemic. As shown in Table 2 above, at the same time the testing capacity increased to meet the needs for planning the academic year as well as taking appropriate measures to identify the new COVID-19 variants were discovered.

**Table 2:** Testing Policies in Germany, Saudi Arabia, UAE, and United Kingdom.

**Country**	**Open public testing policy introduced**	**Current testing policy status, as of 15 Nov. 2020**
Germany	29 April 2020	Open public testing
Saudi Arabia	27 May 2020	Open public testing
UAE	13 April 2020	Open public testing
United Kingdom	N/A – UK's policy is to test anyone showing COVID-19 symptoms	Testing of anyone showing COVID-19 symptoms

*Source: Oxford Covid-19 Government Response Tracker (OxCGRT)*.

As mentioned earlier, the treatment of positive cases in the UAE consists of home isolating of positive cases, in combination with quarantine hotels, and designated field hospitals, as well as highly specialized hospital care depending on the clinical symptoms. To date, a small number of clinical studies have focused on the clinical treatment of COVID-19 cases in the UAE and the outcomes. One of these studies, conducted in the Emirate of Abu Dhabi, described the clinical characteristics of 9,390 patients treated by the public health system in Abu Dhabi over a 3 month period (March–May 2020) (13). The mean age of the patient cohort was 41.8 years, very similar to the UAE population but lower than globally reported figures.

**Figure 3:**
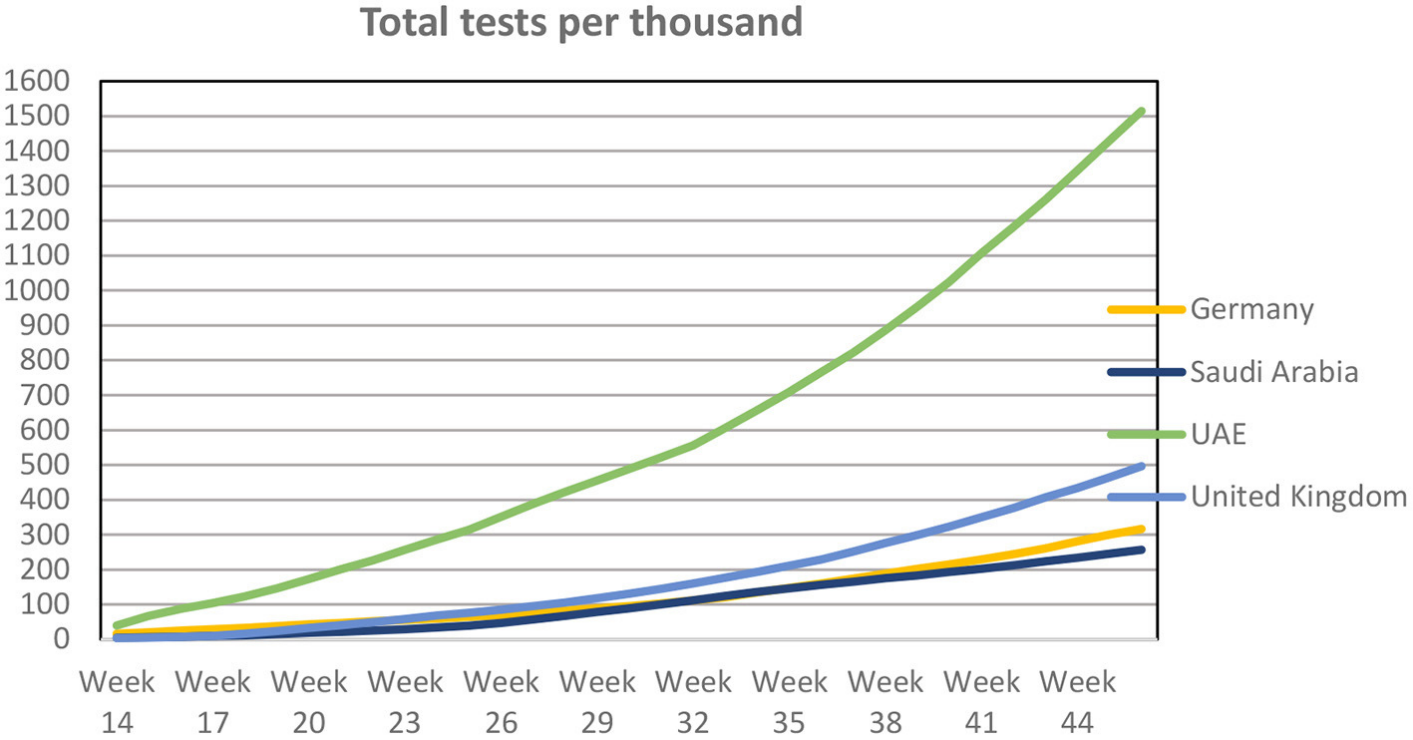
Total number if COVID-19 tests per thousand inhabitants. Source: Our World in Data.

In order to compare the impact of the UAE's mass testing policy, we selected three countries with a similar governmental mass testing (open to the entire population) response to the pandemic and one country (UK) with a testing policy focused on anyone showing symptoms of the disease, as outlined in Table 2 and Figure 3.

The total number of COVID-19 tests has consistently been high in the UAE when compared to the other three countries. At the start of the pandemic (week 15, 12–18 April) the UAE conducted 12 times as many tests per thousand inhabitants (68 vs. 5.4) when compared to the UK. During the same week, the UAE conducted more than treble the number of tests per thousand when compared to Germany (68 vs. 21). Over the subsequent weeks and months, the pattern remained the same. In week 46 (9–15 November) the UAE performed three, five and six times the number of tests per thousand inhabitants compared to the UK, Germany and Saudi Arabia, respectively.

**Figure 4:**
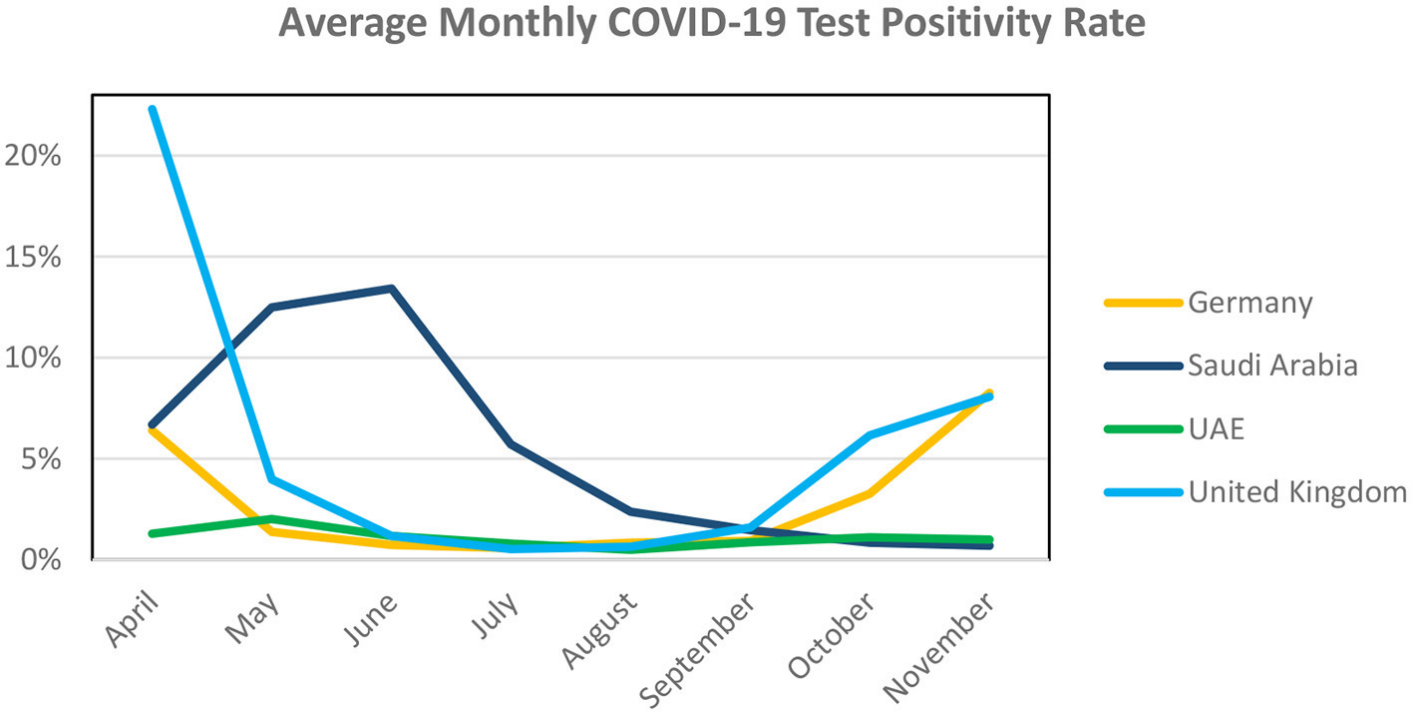
Average monthly COVID-19 test positivity rates. Source: Our World Data.

At the same time, the positivity rates in the UAE (Figure 4) remained relatively stable, around 1%, whereas the rates fluctuated a lot more in the other three countries.

Finally, the number of COVID related deaths has remained low in the UAE when compared to other countries. For example, in November 2020 only four OECD countries performed better than the UAE in terms of deaths per million: Australia, New Zealand, Japan, and South Korea.

As Table 3 above indicates, out of the four selected countries, the overall mortality rates for the three countries with a mass testing policy (Germany, Saudi Arabia, and the UAE) was significantly lower than the country with testing program focused on testing symptomatic cases.

**Table 3:** Week 46: total COVID-19 testes per thousand and deaths per million.

**Country**	**Total test per thousand (week 46)**	**Deaths per million (week 46)**
Germany	316	150
Saudi Arabia	257	162
UAE	1515	54
United Kingdom	496	766

*Source: Our world in data*.

## Discussion

Whilst at the time of the UAE's government decision to embark on mass testing there was limited evidence supporting a mass testing policy, subsequent evidence found that this mass testing approach adopted by the UAE may have resulted in a lower than expected mortality rates (26).

Recent studies found evidence of a statistically significant association between higher COVID-19 mass testing strategies and lower COVID-19 mortality rates: Liang et al. (26) found that 10 additional test per 1,000 people was associated with a 8% reduction in mortality rate, even adjusting for all other factors.

In addition, there is research evidence of mass testing as an effective mechanism to reduce and contain the spread of the virus (27). Mass testing programs, contact tracking and isolation in South Korea contributed to early infection control (28). However, international agencies such as the European Centre for Disease Control and Prevention (ECDC) (29) have expressed concerns about the financial viability of mass or population-wide testing and warned that the testing approach could compromise accessibility or cause delays to the testing of symptomatic cases. The WHO's Interim Guidance (12) recommends that countries adopt a policy of testing symptomatic cases rather than asymptomatic cases.

On 15 November 2020, the UAE had the second highest number of COVID-19 tests per capita and the only country that had conducted more tests was Luxembourg with 1,973 tests per 1,000 inhabitants. In comparison, Germany, Saudi Arabia, and the United Kingdom had rates of 316, 257, and 496, respectively.

It is notoriously difficult to assess the impact of various health system have on the population health outcomes (10). The Oxford COVID-19 Government Response Tracker (OxCGRT) developed by researchers from the Blavatnik School of Government at the University of Oxford (6) provides a standardized classification of 18 policy measures governments can take to deal with the pandemic, including measures such as face coverings, testing, contact tracing, and school closures. Whilst it may be impossible to analyse the impact of each of the 18 policy measures, this research indicates that a testing policy focused on mass testing symptomatic and asymptomatic cases may have a strong impact on the ultimate outcomes of concern.

## Conclusions

Previous studies (8, 10) have reviewed the ambition and commitment of the UAE to build a world-class health system and found some evidence of improved outcomes from the health system reforms in the UAE. This study shows additional evidence to support the ongoing health system reforms as it relates to the mass testing program in the UAE. The UAE has conducted one of the highest numbers of COVID-19 tests in the world leading to the positive impact of mass testing on tracing and isolation, i.e., containment of the virus, as well as deaths.

Despite the highly contagious nature of the COVID-19 disease, variation in government responses in dealing with the pandemic has had a large impact on the ultimate outcomes of interest from a public health perspective. There is an urgent need to ensure governments learn from other governments to successfully contain and manage public health outbreaks such as COVID-19 disease pandemic.

## Author Contributions

FA-H, SA-Ma, SA-Me, ZA-Y, MSP, and EK: conceptualization. MSP and EK: methodology, writing—review, and editing. EK, MSP, and SA-Me: validation. EK: formal analysis, writing—original draft preparation, visualization, and supervision. EK, MSP and SA-Ma: project administration. All authors have read and agreed to the published version of the manuscript.

## Conflict of Interest

The authors declare that the research was conducted in the absence of any commercial or financial relationships that could be construed as a potential conflict of interest.
